# Supplementary material for: A set of multi-entry identification keys to African frugivorous flies (Diptera, Tephritidae)
Source: Zookeys. 2014 Jul 24;(428):97–108. doi: 10.3897/zookeys.428.7366 (PMC4143993; doi:10.3897/zookeys.428.7366)
Supplement: Supplementary material 6 — Key to Ceratitis [file zookeys-428-097-s006.zip › SF6_ZooKeys_key to Ceratitis/key/SF6_ZooKeys_key to Ceratitis/Media/Html/Ceratitis scaevolae .htm]

Ceratitis (Ceratalaspis) scaevolae (Munro)


***Ceratitis*** **(*Ceratalaspis*)
*scaevolae* (Munro)**

*Pardalaspis scaevolae* Munro, 1929: 394.

Body
length: 5.14 (4.60-6.25) mm; wing length: 4.36 (3.85-4.75) mm.

Male

Head.
Antenna yellow to yellow-orange. Third antennal segment twice as long as second
segment. Arista bare. Frons with short scattered hairs which are largely the
same colour as the frons; convex, in lateral view slightly projecting forwards
at antennal implant. Face usually yellow-white. Chaetotaxy normal for subgenus.

Thorax.
Postpronotum white, unspotted. Ground colour of mesonotum pale with slight
orange tinge. Chaetotaxy normal for subgenus. Scapular setae usually black,
sometimes yellow-reddish (inner) to reddish black. One anepisternal bristle.
Scutellum white, with three black markings joined apically; basally no spots.
Subscutellum completely dark.

Legs
yellow; setation typical for subgenus, mainly pale especially on femora.
Posterior and posterodorsal rows on fore femur darkish.

Wing
bands with markings extensively yellow to yellow-brown. Banding, marginal band
continuous; cubital band joined with discal band; medial band absent; crossvein
r-m well beyond middle of discal cell, vein R1 before crossvein. Crossvein
dm-cu posterioly more outwards than anteriorly.

Abdomen.
Yellow colour. Setation and banding typical for subgenus.

Female

As
male except for the following characters: Posterior and posterodorsal rows on
fore femur sometimes less dark; scutellar spots occasionally joined together,
forming one large semicircular spot. Oviscape shorter than abdominal terga 3-6
combined.

(Description
after De Meyer, 1998)
